# Monitoring of Serological, Cellular and Genomic Biomarkers in Transplantation, Computational Prediction Models and Role of Cell-Free DNA in Transplant Outcome

**DOI:** 10.3390/ijms24043908

**Published:** 2023-02-15

**Authors:** Víctor Jimenez-Coll, Santiago Llorente, Francisco Boix, Rafael Alfaro, José Antonio Galián, Helios Martinez-Banaclocha, Carmen Botella, María R. Moya-Quiles, Manuel Muro-Pérez, Alfredo Minguela, Isabel Legaz, Manuel Muro

**Affiliations:** 1 Immunology Service, Instituto Murciano de Investigación Biosanitaria (IMIB), Hospital Clínico Universitario Virgen de la Arrixaca (HCUVA), 30120 Murcia, Spain; 2 Nephrology Service, Instituto Murciano de Investigación Biosanitaria (IMIB), Hospital Clínico Universitario Virgen de la Arrixaca (HCUVA), 30120 Murcia, Spain; 3 Department of Legal and Forensic Medicine, Biomedical Research Institute (IMIB), Regional Campus of International Excellence “Campus Mare Nostrum”, Faculty of Medicine, University of Murcia, 30100 Murcia, Spain

**Keywords:** human leukocyte antigen (HLA), donor-specific antibody (DSA), monitoring biomarkers, kidney transplantation, chronic rejection, regulatory cell, cfDNA, cell-free DNA, long-term graft survival, computational prediction

## Abstract

The process and evolution of an organ transplant procedure has evolved in terms of the prevention of immunological rejection with the improvement in the determination of immune response genes. These techniques include considering more important genes, more polymorphism detection, more refinement of the response motifs, as well as the analysis of epitopes and eplets, its capacity to fix complement, the PIRCHE algorithm and post-transplant monitoring with promising new biomarkers that surpass the classic serum markers such as creatine and other similar parameters of renal function. Among these new biomarkers, we analyze new serological, urine, cellular, genomic and transcriptomic biomarkers and computational prediction, with particular attention to the analysis of donor free circulating DNA as an optimal marker of kidney damage.

## 1. Introduction

The process and evolution of an organ transplant procedure has evolved in the prevention of immunological rejection with the improvement in the determination of immune-response genes, including more important genes, more polymorphism detection, more refinement of the response motifs, the analysis of epitopes and eplets, and PIRCHE (predict indirectly recognizable HLA epitopes). These advancements are capable of fixing complements and post-transplant monitoring with promising new biomarkers [1,2,3] and surpassing the classic serum markers, such as creatine and other similar parameters of organ function [4,5,6,7,8,9,10,11,12,13,14,15]. 

**Figure 1:**
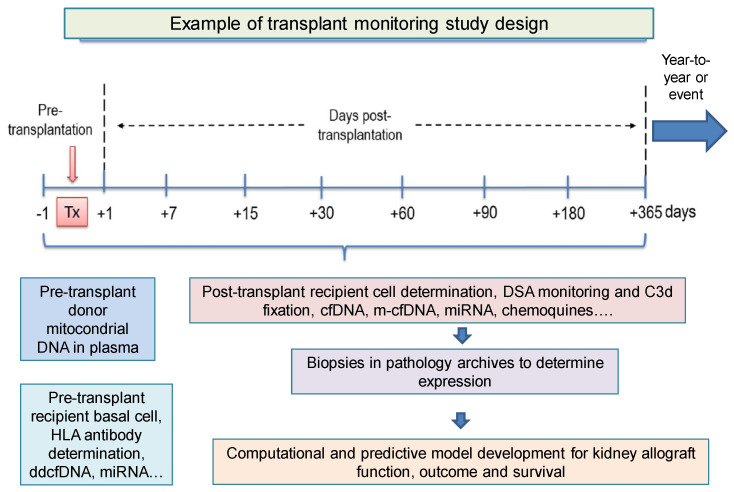
Biomarkers in allotransplantation with a virtual example of hypothetical promising biomarkers and their eventual pre- and post-transplant monitoring time. cfDNA, cell-free DNA; ddcfDNA, donor-derived cell-free DNA; DSA, donor-specific antibodies; HLA, human leukocyte antigens; miRNA, microRNA. C3d: Final degradation product of the third component of complement (C3). m-cfDNA: mitocondrial cfDNA.

The situation can be improved in the specific case of kidney transplantation (KT), performed to cure end-stage kidney disease. Despite the improvement of immunosuppression with better approaches, better induction and better prevention and treatment options, the appearance of problems post-transplantation, especially subclinical rejection (SCAR), which is more difficult to detect at the time (some authors find rates of 30% diagnosed with biopsy [16]) is still a challenge. It is crucial to favor the control and follow-up of the evolution of the transplant recipient to find new noninvasive biomarkers for the early diagnosis of eventual graft injury, which should be easy to manage, specific, reproducible and clinically validated. A basic list of these hypothetical promising biomarkers and their tentative monitoring times is shown in Figure 1.

Because patient survival is dependent on multiple risk factors, including the severity of the recipient’s primary disease, HLA antigen matching between the recipient and donor and the presence of donor-specific antibodies (DSA) in the recipient that were not detected pre-transplant or developed de novo (dnDSA), as well as other confounding factors related to the donor, the lack of rigorous and accurate real risk assessments makes correct post-implantation follow-up even more difficult. The interaction between the risk factors of the recipient and the donor makes the prognosis of transplant recipients one of the great pending subjects in current transplantology.

Among these new biomarkers, we will analyze and discuss new serological, urine, cellular, genomic and transcriptomic biomarkers and computational prediction, with particular attention to the analysis of donor-derived cell-free DNA (ddcfDNA) [17,18] as an optimal marker of kidney damage, and discussion their limitations and future directions.

To avoid possible biases inherent to our experience in this field, the selection of biomarkers included in our article was based on a rigorous and exhaustive analysis of publications in the scientific literature on transplant monitoring, with various search terms related to this matter, such as “transplant”, “biomarkers”, “monitoring”, “rejection”, “graft survival”, “graft injury” and “graft fibrosis.” Only those biomarkers with more than one report in the literature were finally selected.

## 2. Serological and Urine Biomarkers

### 2.1. Classical Markers

Aside from physical examination, we have traditionally used routine serological or urinary markers such as urine volume, evaluation of albuminuria or proteinuria, determination of serum creatinine, estimation of glomerular filtration rate (eGFR) based on serum creatinine and HLA antibody profiling to assess the absence or presence of DSA when a kidney is transplanted. Serum creatinine levels, which rise late in the lesion and are unrelated to the type of lesion, are the most commonly used biochemical parameter [15]. However, serum levels of this parameter are neither sensitive nor specific for determining graft status [16]. Furthermore, serum creatinine levels are neither specific nor predictive when predicting or evaluating the progression of chronic lesions [15]. The gold standard diagnostic test for determining transplanted kidney rejection is a kidney biopsy, which can reveal chronic immune injury, interstitial fibrosis and tubular atrophy [17]. Because of the heterogeneity of processes underlying the same lesion, biopsies have low sensitivity and specificity, a lack of standardization and quantitative thresholds, and even sampling errors [19]. Due to the procedure’s high invasiveness, researchers are looking for more effective immune monitoring or imaging techniques for accurate diagnosis [20,21,22].

For several years, attempts have been made to reduce rejection rates by meticulously serologically monitoring the specificities and titers (via mean fluorescence intensity (MFI)) of anti-HLA antibodies, including their identification with high-resolution tests (single antigens) or the ability to fix complements (C1q and C3d assays) in conjunction with other non-HLA antibodies (ETAr, ATR1,...), and, more recently, new biomarkers for potential use in clinical practice [23].

### 2.2. New Markers

New serological and urinary markers may seem essential for evaluating renal function post-transplant. Among these, several can indicate damage or renal graft function. Some of the biomarkers that we will analyze in this review are:

Neutrophil gelatinase-associated lipocalin (NGAL). Neutrophils secrete NGAL during an inflammatory response and it functions as an acute-phase protein. NGAL levels in urine (uNGAL) can also be measured; siderophores and metalloproteinase 9 are its primary ligands and markers of acute tubular cell injury [24]. NGAL can also be used to detect kidney damage [25]. The creatinine concentration in urine and plasma rises about 2 h after renal cell destruction [26]. In contrast, NGAL can assess the transplant status a few hours after surgery. Other researchers have discovered that uNGAL was lower in renal recipients who did not have reperfusion injury on the first day after implantation [27]. A decrease in these protein levels on day three was also a good predictor of renal function one month later. Another study [24] concludes that uNGAL may be more helpful in assessing renal function in the first week after KT when combined with other markers. Another study found that NGAL levels in urine and serum could be used to predict kidney damage and as a biomarker of acute kidney injury after transplantation [28]. According to the researchers, patients with acute kidney injury had higher uNGAL levels than patients without acute kidney injury, with a 2 h post-transplant increase. A different group of researchers [29] discovered a significant increase in sNGAL levels on day one in HLA-incompatible KT recipients who developed rejection within a month, demonstrating that uNGAL was the most sensitive marker to detect acute KT dysfunction in a living donor. Finally, according to other authors [30], sNGAL and NGAL/creatinine can be used to estimate kidney and transplant function change.

Kidney injury molecule-1 (KIM-1). KIM-1, also known as T cell immunoglobulin mucin receptor 1 (TIM-1) and found in pathology in the kidney, liver, liver and spleen [31], is involved in T and B cell biology. KIM-1 is thought to aid in diagnosing kidney disease because its extracellular domain is cleaved by metalloproteinases and secreted in urine [32]. According to the FDA [33], the protein is a biomarker of kidney damage caused by nephrotoxic drugs. KIM-1, like NGAL, is found in urine 24 h after exposure to various induced nephrotoxic factors, and its concentration has been shown to influence eGFR values and thus predict kidney damage [34], though other authors disagree [34]. KIM-1 seems to be a good predictor of KT rejection [25]. As a result, another study [35] suggests that sKIM-1 could be used to predict renal failure early in the rejection process. They also discovered that osteopontin (OPN) and sKIM-1 improved the prediction accuracy. Other researchers [36] studied uKIM-1 mRNA expression and urinary and serum KIM-1 proteins in renal recipients with rejection and chronic dysfunction and concluded that KIM-1 could be used to monitor renal recipients, which can help diagnose AR and chronic dysfunction and be an independent factor for predicting transplant loss.

CXCL-10. It is a chemokine secreted by renal graft leukocytes that regulate angiogenesis in conditions such as wound healing, ischemia and neoplasia [37]. It is also a sign of inflammation. CXCL-10 (uCXCL-10) in urine appears to be more sensitive and specific than creatinine in serum [37]. Its levels can aid in the detection of early signs of acute renal failure as well as the diagnosis of noninvasive kidney disease [38]. Many studies on the role of CXCL-10 in renal rejection have been conducted worldwide. According to some authors [26], uCXCL-10 is well-identified in ACR and correlates with plasma creatinine levels. Other studies [39] found that measuring urinary CXCL-10 and creatinine levels, then calculating the ratio of these two parameters, can accurately predict the risk of AMR. Other researchers [40] think children’s CXCL-10/creatinine ratio is a promising biomarker of acute cellular rejection. CXCL-10 mRNA detection in urine [41] has also been proposed as an ideal biomarker of biopsy-confirmed rejection. Other authors believe that measuring these chemokine levels prior to transplantation is critical because high levels in the serum pre-transplant indicate a high risk of rejection and transplant failure [23]. Another study discovered that CXCL-10 serum levels greater than 150 pg/mL prior to transplantation predispose to severe rejection [42], and others discovered that CXCL-10 levels in urine could rise in acute rejection and BK virus infection. This chemokine is unable to differentiate between these conditions [43]. According to another group, CXCL-10 levels rise with BK virus replication and infection-related nephropathy [44].

Cystatin C (CysC). It is a cystatin superfamily proteinase inhibitor or cysteine protease inhibitor that primarily inhibits cathepsins L, B and H and is required for intracellular protein and peptide catabolism [45]. The glomeruli freely filter this protein, reabsorption occurs through reflux and catabolism occurs in the renal tubules [46]. When the renal tubules are damaged, cystatin appears in the urine [47]. It is thought to occur two days before elevated creatinine levels in patients with end-stage renal disease [48]. In acute renal failure, CysC is an excellent marker of renal function, especially as renal function deteriorates and rejection occurs. Other authors [49] have found that determining CysC 14 days after transplantation outperforms creatinine in terms of sensitivity and specificity.

Osteopontin (OPN). BSP-1 (bone sialoprotein) and SPP-1 (secreted phosphoprotein 1) are other names for OPN. OPN, like cytokines, regulates the immune system and is involved in tissue and bone remodeling, inflammation, atherogenesis, cell survival and kidney damage [50]. It is crucial in the development of chronic inflammatory diseases and cancer. Based on plasma, the highest urinary OPN can predict renal function deterioration and estimate the risk of cardiovascular death [51]. Urinary OPN, like NGAL and KIM-1, is a promising biomarker for detecting renal damage in neonates, according to other researchers [52]. It appears to be a promising biomarker in KT rejection because of its critical role in the inflammatory process [53]. Other studies have found higher levels of this protein in KT with AR biopsies [54], and one group believes that OPN levels in plasma predict the severity of ACR in renal recipients. The diagnostic findings corroborated the changes in biopsy age [55].

Clusterin (CLU). Low levels of CLU impair renal function in ischemia–reperfusion disorders by destroying kidney tissue and increasing cell apoptosis [56]. CLU is involved in both the apoptotic and antiapoptotic processes. Other authors [57] argue that it adds nothing new to the discussion. Another study [58] found that urinary CLU could be a useful noninvasive marker for detecting renal damage in children with systemic lupus erythematosus, which predisposes them to end-stage organ failure. CLU was discovered to be a marker of sublethal kidney damage in another study of children undergoing allogeneic stem cell transplantation [59]. Finally, CLU may be an important biomarker when KT function is delayed, with levels rising as early as 4 h after surgery [60].

CXCL13. It is a chemoattractant necessary for forming the germinal center (GC) and alloantibodies. Serum CXCL13 levels can be correlated with HLA antibody formation post-transplantation. With a murine skin graft model, an author co-cultured in vitro follicular helper T cells (Tfh): human B cells to assess CXCL13 production by human lymphocytes in recipients with and without de novo DSA. They found that CXCL13 was detectable in the blood of allografted mice and correlated with B, Tfh and GC cell responses [21] and also observed increased expression of CXCL13 in the draining lymph nodes of allografted mice compared with recipients of syngeneic grafts or without previous treatment. The serum levels also preceded the detection of post-transplant DSA. Similarly, Tfh–human-B-cell interactions, which are very important in plasmablast differentiation and IgG formation, also showed CXCL13 expression. CXCL13 levels in recipients with de novo DSA were higher than in stable recipients, presenting CXCL13 as a potential biomarker for HLA antibodies.

In the same way, another chemokine, CXCL9, has also been implicated in AMR in KT [28].

## 3. Cellular Biomarkers

### 3.1. Classical Markers

The classic cellular measurements include the percentages of particular populations, levels of certain molecules in the membrane, the expression of costimulatory molecules (CD28, CD69, CD95...) and the production of soluble or intracytoplasmic cytokines. Many groups, ours in particular, classically have had much experience helping to establish models of these methods, especially in renal, hepatic and cardiac transplantation [26,27,29,31,61,62,63,64].

A procedure that many transplant hospitals routinely incorporate to monitor rejection, infection and immunosuppression based on a cellular-metabolic approach is the ImmuKnow assay [26] (Cylex), which is the only FDA-approved test to assess changes in ATP production by CD4+ T cells over time, a potential marker of receptor status. The results that depend on the activation threshold of CD4+ T cells are reported as usual, high (indicating low immunosuppression and risk of rejection) or low (suggesting excess immunosuppression).

### 3.2. New Markers

Regarding biomarkers of cellular response, the latest trends in the last ten years are more directed at cells with an eventual regulatory function rather than an effector one per se.

In this way, Tregs lymphocytes, Bregs cells, follicular Th cells (meaningful in the production of antibodies) and myeloid suppressor cells have dominated the transplantation publications in this cellular section in recent years [8,9,24,26,65].

On the other hand, recent studies show a predominance of a Th2 response in grafts with accommodation as opposed to the classic Th1 response in grafts with rejection, and there are also studies in which the presence of regulatory T cells (Tregs) in transplant recipients has been studied in the renal and hepatic fields [24,65]. As previously mentioned, the most referenced is the monitoring of Tregs and Bregs cells post-transplantation, which is an option to optimize transplantation, with interesting publications on the determination of B cell clusters, with an essential role in transitional B cells [26]. These cells modulate the immune response predominantly through IL-10 and possibly BAFF (B cell Activating Factor)-dependent mechanisms [32].

In this sense, hypothetically, sensitized patients who induce these regulatory cells could be considered low-risk and be subjected to a lower level of clinical immunosuppression to avoid its complications over time.

However, there are other cells recently reported as crucial in producing antibodies, such as Tfh cells. They are a subclass of lymphocytes specialized in assisting B cells to produce antibodies in circulating B cell follicles (cTfh) in co-blood [31]. These cTfhs have been associated with developing de novo anti-HLA antibodies (dnHLA) and de novo DSA and with acute and chronic allograft rejection [11].

Bregs appear to prevent the development of Tfhs by expanding follicular Treg cells and inhibiting the Tfh-mediated differentiation of plasma cells in vitro [64]. In this sense, specific imbalances in renal transplantation have been reported among these populations [63].

In addition, many B cell differentiation molecules are also important in these processes, as recently reported [33]. Thus, BLYS-BAFF modulates the survival and proliferation of B cells through three receptors: BR3/BAFF-R, TACI and APRIL. This one also joins BCMA. In addition, BAFF activates NFkB by binding to BCMA and TACI and increases the expression of Bcl-2, inhibiting cell apoptosis [16].

Most of the cellular and molecular pathways converge on molecules common to pathways of activation and proliferation or suppression and apoptosis. These essential molecules are involved in the differentiation, maturation and activation of B cells to produce antibodies.

## 4. Genomic and Transcriptomic Biomarkers

### 4.1. Classical Markers

In recent years, the biomedical research community has attempted to respond by leveraging the power of omics platforms and generating big data, allowing for the measurement and analysis of large-scale molecular signatures from tissue biopsies and circulating cells [33,65,66,67]. Despite its small size, it can boast two predictive tests, the AlloMap [68] and AlloSure [69] assays, and other assays found in clinical trials, such as the Signatera^TM^ and Prospera^TM^ trials. Other novel noninvasive organ evaluation tools have recently been proposed, such as donor plasma mitochondrial DNA (mtDNA), which can be easily tested prior to transplantation and may be a promising predictive biomarker for delayed graft function (DGF) [70]. Six months after RT, the linear prediction model, which included mtDNA in plasma, creatinine in donor serum and warm ischemia time, demonstrated a high noninvasive predictive value for DGF reduction and graft function, as well as a correlation with graft survival. 

### 4.2. New Markers

The first example of how transcriptomics can improve transplant precision medicine is the AlloMap gene expression profiling test. AlloMap was approved as a Class II Medical Device by the FDA in 2008. In the absence of endomyocardial biopsy support, it is a blood test that analyzes and quantifies the gene expression levels of a panel of 11 genes in peripheral blood mononuclear cells, yielding a score that can categorize cardiac recipients as having a higher or lower risk or probability of developing ACR. In stable patients between 6 months and five years after heart transplantation, this AlloMap assay can rule out the presence of grade 2R or greater acute cellular rejection [71].

TruGraf^®^ (Transplant Genomics, Framingham, MA, USA), Viracor TRAC^®^ (Eurofins Viracor, Lenexa, KS, USA), OmniGrafTM (Transplant Genomics, Framingham, MA, USA) and QSant (Nephrosant, San mateo, CA, USA) are some of the other assays used in biopsy detection techniques [72]. Three months after the transplant, TruGraf^®^ detects differential gene expression in peripheral blood to monitor recipients with stable renal function and to guide immunosuppressive therapy optimization in hepatic recipients. Other clinical trials, such as INTERCOMEX (NCT0129 9168) conducted prospectively by the Alberta Transplant Applied Genomics Center (ATAGC), have produced promising results, opening a new paradigm in biopsy evaluation. Using blood RNA sequencing in kidney recipients enrolled in the Genomics of Chronic Allograft Rejection (GoCAR) prospective cohort study, a panel of 23 genes was identified to assess the risk of pre-kidney-transplant rejection and customize the immunosuppressive regimen [73]. A new noninvasive assay that detects a specific panel of metabolites in urine can predict kidney graft rejection in a multicenter prospective observational study (PARASOL) [74]. Another prospective study in multicenter cardiac recipients used histopathology, immunostaining, DSA antibodies at the time of biopsy and microarrays to examine graft gene expression [75]. Tissue-based analysis of pathogenic transcripts expressing NK cell, endothelial cell, macrophage activity and INF effects allowed for accurate AMR classification and quantification of injury and disease activity [75].

On the other hand, several articles point to the role of miRNA expression in kidney transplantation and suggest their role in acceptance or rejection and their utility as biomarkers [76]. 

Several miRNAs have been identified in biopsies or blood mononuclear cells of patients with AMR in renal RT [48]. Thus, post-KT miRNA deregulation has been reported [77], as has the existence of miR-142-3p overexpression in blood in patients with operational tolerance. This observation reinforced the hypothesis that miR-142-3p might play a regulatory role in T cells by controlling leukocyte activation. In addition, miR-155 contributes to Rituximab resistance by inducing cell-survival signals [76]. Naturally, other phenomena could occur after transplantation, including ischemia and reperfusion, cellular rejection and disease recurrence, and miRNAs could be associated with these phenomena. Thus, in KT, miR-142-5p demonstrated its presence in chronic AMR and is overexpressed in ACR biopsies, and miR-142-3p was associated with interstitial fibrosis and tubular atrophy in urine, and miR-338-5p in serum [76]. We also recently published studies of microRNAs in KT [34]. We detected decreased miR-150-5p, and with computational prediction, we designed an interaction–repression model of this miR150-5p with the methyl-CpG binding domain protein 6 (MBD6), as well as proteins that are regulated by it, such as MBDA, ASXL2, FOXK2, KDM1B, BAP1 and HCFC1. On the other hand, the presence of free circulating DNA (cfDNA) has been described in transplant rejection, with remarkable impact in recipients of heart, kidney, liver and recently lung transplants [19,35], where it can become a noninvasive test of rejection, infection and immunosuppression. We will expand on this phenomenon as an isolated epigraph in the last section of this review of biomarkers.

Lastly, epigenetics has also been associated with post-transplant evolution with DNA methylation phenomena regulating processes and pathways involved in its evolution [36,37]. Surely this field will be especially novel for future studies.

## 5. Computational Prediction Biomarkers

### 5.1. Classical Markers

There are few “classical” approaches to computational prediction biomarkers; among them are Rene Dusquesnoy’s HLAmatchmaker tools [5] and Histocheck in the case of bone marrow transplantation [78], although they are somewhat indirect measures. The Predicted Indirectly Recognizable HLA Epitopes (PIRCHE-II) algorithm can also be used to calculate the number of theoretical T cell epitopes available for donor–recipient combinations for indirect allorecognition [8]. These are the pioneers of computational transplantation prediction. In any case, the first goal should be to develop personalized predictive models that consider both the giver’s and the recipient’s unique characteristics. The recent adoption of electronic health records (EHR) and artificial intelligence (AI) in organ transplant medicine is encouraging, as there are a variety of data-driven workflows available to build such predictive models from clinical data [79]. Aside from AI, recent advances in NGS indicate the possibility of discovering new biomarkers reflecting immune-related mechanisms underlying graft failure and acute and chronic rejection events [13].

Computational models in kidney transplantation can be used to explore different scenarios and optimize the outcome of kidney transplantation [80,81]. These models can help improve the effectiveness of patient selection, evaluate the risk of complications and improve the long-term outcomes of kidney transplantation [80]. Models can also be used to identify potential improvement opportunities in the current transplantation process, such as improving the matching of donors and recipients, maximizing the use of available resources and optimizing the timing and sequencing of transplantation [82]. The proposed computational models are not exclusive to renal transplantation; currently, the field of application in biomedicine is vast [83,84].

### 5.2. New Markers

An important point is the lack of noninvasive SCAR biomarkers for KT recipients since it can only be diagnosed by protocol biopsy and is correlated with worse KT outcomes. In this sense, there are investigations to study these processes that are so difficult to diagnose. There are articles with simulation, computational prediction and managing Gene Expression Omnibus (GEO) databases as training and validation cohorts. Subsequently, stepwise logistic regression methods can be applied to build a particular genetic signature of each process and/or study group for any critical event in transplantation that we want to analyze [10,85]. The obtained biomarkers can be filtered using automatic learning algorithms, resulting in genes that can be extrapolated to the cell populations in question, as well as develop and validate a new noninvasive signature of the genes obtained to diagnose SCAR or any other event as potential tools for clinical practice and to perform a timely intervention, as suggested [66]. Interactions can be represented graphically using nodes (circles) representing components (genes, proteins, metabolites, etc.) and edges (links) representing interactions of physical/regulatory connections between two nodes [19,35]. Several recent publications [19,86] have covered and reviewed the fundamental principles of network medicine, the network’s main topological measures (e.g., node degree, betweenness centrality and closeness centrality) and examples of experimental pipelines and their potential clinical utility. Innovative interaction-based approaches that link these molecular signatures obtained by different groups with pathology development and clinical events to demonstrate that potential new biomarkers are targeted to networks useful for the diagnosis and classification, stratification, and prognosis of various pathologies that disable the organ in question and whose only terminal treatment is organ transplantation are successful examples. These pathologies include atherosclerosis, hypertrophic cardiomyopathy, chronic kidney disease, chronic liver disease and cancer [56,87,88]. In any case, graft rejection remains a significant cause of complications, and these preliminary studies can help use these GEO databases to identify potential new drug targets, as suggested by our group and others [34,89,90,91]. Researchers discovered that a panel of six core genes, including DOCK2, NCKAP1L, IL2RG, SLAMF8, CD180 and PTPRE, were upregulated in AR vs. NAR patients and correlated with IFN response and inflammatory response using the Weighted Gene Coexpression Network Analysis (WGC-NA) algorithm to analyze microarray data [8].

Furthermore, the WGCNA algorithm was used to identify rejection-related modules and core genes in three KT-related gene datasets (GSE46474, GSE15296 and GSE14067 from GEO) [85]. CD200R1, VAV2, FASLG, SH2D1B and RAP2B have been identified as potential network-oriented biomarkers for detecting post-KT rejection [89]. By integrating transcriptomic and metabolomic signatures of biological samples isolated from liver transplant recipients, a WGCNA-derived PPI network predicted that overexpression of pyruvate kinase L/R could significantly impact transplant prognosis [90].

The application of interactions promises to be ideal for identifying rejection with these new potential biomarkers or drug targets and allow us to discard the hackneyed hypothesis of a “gene interaction” disease [26,66]. Finally, we must work on undefined bioinformatic processes such as analyzing interest interactions, obtaining problem genes of a specific pathology using online databases and previous bibliography, defining modules, enriching hundreds of pathways and molecular mechanisms and clinical validation, estimation and prediction of the obtained results [92,93]. The STRING database [94] and DisGeNET, which contain genes associated with human pathologies, can also integrate known and expected physical/functional interactions between proteins. One strategy would be to use Cytoscape, an open-source software platform, to generate a graphical representation of key factors, significant nodes and molecular pathways that could benefit from further experimental validation [91]. As a network medicine paradigm, it would be advantageous to conduct a comprehensive multiomic analysis of graft recipients’ blood cells or a biopsy before and after transplantation to determine which genes and/or pathways can be predictive of clinical events and to design prospective multicenter studies to consolidate these computational markers for clinical event prediction in transplantation [38,85,91].

## 6. Cell-Free DNA Biomarkers 

### 6.1. History

DNA micro-chimerism, or the mixing of donor and recipient cellular material during transplantation, was discovered for the first time in the 1990s [95]. Lo et al. [96] isolated Y-chromosome genetic material from female transplant recipients’ circulating plasma in 1998, progressing from probe-based cytostaining and cell homogenates to detecting cell-free DNA. It was the first time donor-specific cell-free DNA (ddcfDNA) was found in a transplant and an early indication that accurate donor genotype assumptions could be beneficial. Molecular methods made a breakthrough in distinguishing donor and recipient DNA, initially focusing on HLA-specific quantitative PCR. The technique’s ability to distinguish between HLA-compatible recipient–donor pairs was limited [97]. Following that, detecting ddcfDNA with microfluidic digital PCR proved helpful in accurately detecting differences in SNP frequencies between donor and recipient [92], but complete donor/recipient genome sequencing, resources and a significant time commitment were required. The development of population-level underestimation of allele frequencies derived from the Human Genome Project was the next significant step toward ddcfDNA becoming a clinically valuable tool in transplantation [26,31]. It was then demonstrated that they could be obtained without requiring complete donor genotyping [64]. In the same year [63], the feasibility of quantifying ddcfDNA without a complete recipient or complete donor genotype was demonstrated by interrogating only 266 SNPs. In the United States, there are currently three commercially available ddcfDNA assays for clinical use in renal transplantation: AlloSure from CareDx, Prospera from Natera and TRAC from Viracor Eurofins [96]. The main difference between these tests is the number of SNPs measured [98], which requires comprehensive blood analysis at a centralized laboratory. Increased ddcfDNA predicts AMR better than T-cell-mediated rejection regardless of platform, a poorly understood phenomenon. At validated thresholds, ddcfDNA is more sensitive to antibody-mediated rejection. This could be because a component-activated membrane attack complex is recruited, resulting in cell lysis and the release of additional intracellular debris, including cfDNA [93]. The targeting of endothelial microvasculature can also result in an ischemic environment, which contributes to necrosis. In T-cell-mediated rejection, phagocytosis after apoptosis can sequester more intracellular contents, resulting in less measurable cfDNA despite graft injury. Finally, the absence of ddcfDNA outperforms the presence of ddcfDNA in predicting allograft rejection, which may be one of the reasons for rejection. This discovery has been replicated by several groups [29]. As a result, the primary clinical utility, in keeping with the original intent, is ruling out rejection suspicion and confidently avoiding unnecessary biopsy.

### 6.2. Is It Useful and Where Is cfDNA Analysis Headed?

In a 2020 retrospective study, Goussous et al. [99] linked ddcfDNA elevations to concomitant BK viremia; however, the incidence was not high enough for statistical purposes. Kant et al. [100] investigated the effect of BK viremia and concurrent rejection on ddcfDNA levels. Despite a positive correlation between BK viremia and ddcfDNA levels, rejection did not affect BK viremia patients’ ddcfDNA levels. Although it is thought that cell injury increases ddcfDNA levels, there is currently insufficient research to define the pathophysiology of insults that affect ddcfDNA levels but are not mediated by rejection. Furthermore, because the initial validation studies were conducted when rejection was suspected and a biopsy was performed, the optimal ddcfDNA levels for specific post-transplantation periods are unknown. Several recipient and donor factors interact (for example, DSA and repeat transplant status). Attempts to define a “normal” range of ddcfDNA in transplant recipients with no suspicion of rejection yielded a median value of 0.23%, with a wide range ranging from 0.2% to 1.2% [101]. Because many of these “normal” values are higher than the recommended cut-off points for detecting rejection, the value of ddcfDNA in a recipient with excellent and stable graft function is unknown. As clinical ddcfDNA experience grows, concerted efforts to fine-tune the optimal use of this test should occur. Among them are monitoring the NPV response to chronic rejection treatment, easing immunosuppression regimen transitions and identifying states of graft immunosenescence. Previously, serial ddcfDNA measurements were proposed as a possible adjunct to post-biopsy treatment to monitor the response to rejection treatment [102]. Cedars-Sinai Medical Center researchers are currently studying the use of ddcfDNA (NCT03859388) in assessing the treatment response for chronic antibody-mediated rejection (ABMR). Anti-DSA and a biopsy with histological confirmation are required for the correct diagnosis of ABMR. DSA, serum creatinine and renal biopsies are currently used to assess treatment response [103]. However, only a tiny percentage of ABMR patients will see a reduction in DSA due to treatment. Furthermore, patients with chronic ABMR may have elevated DSA levels despite evidence of normal renal function and biopsy rejection. The study’s recruitment phase is now complete. The findings are set to be published early next year. Other clinical trial data-collection consortiums, such as the CTOT or the iGeneTRAiN program, could help improve those diagnoses [51]. CareDx-funded researchers hope to use ddcfDNA to distinguish between a healthy person’s resting immune status and suspected rejection. The ADMIRAL multicenter observational study (NCT04566055) will validate previously collected clinical trial data to determine the efficacy of ddcfDNA as a predictor of long-term graft survival [34]. Although there was no statistical difference between the non-rejection and biopsy-proven rejection groups in serum creatinine levels, the non-rejection group had significantly higher levels of low ddcfDNA than the biopsy-proven rejection group.

**Figure 2:**
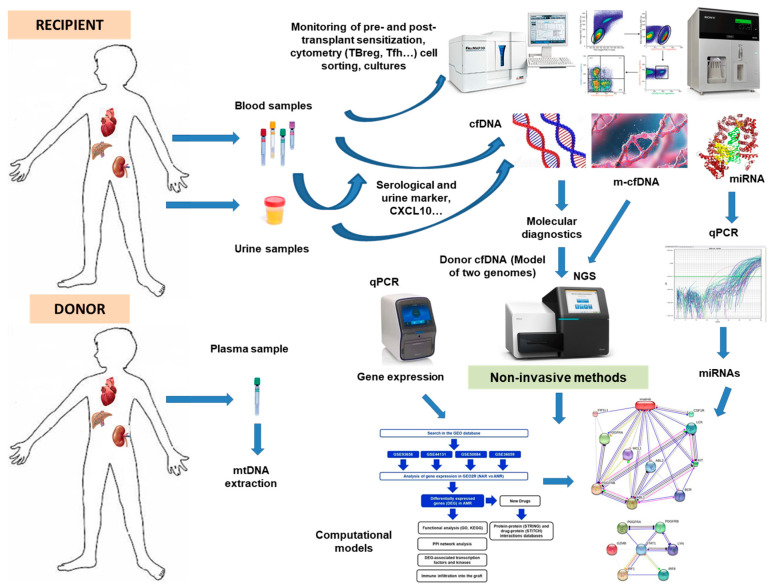
Design of approximate strategies for future biomarkers in donor and recipient pairs in an organ allotransplantation with a small blood sample and/or urine extraction. cfDNA, cell-free DNA; ddcfDNA, derived-donor cell-free DNA; DSA, donor-specific antibodies; miRNA, microRNA; mtDNA, mitochondrial DNA; qPCR, quantitative PCR; NGS, next-generation sequencing; m-cfDNA, mitocondrial cfDNA.

Furthermore, ddcfDNA was linked to the subsequent development of de novo DSA when the threshold was reduced to 0.5%. The preliminary data on ddcfDNA’s utility in early subclinical surveillance and rejection detection are promising. Figure 2 depicts a hypothetical integration scheme of the various approaches we have seen and reviewed as a final point for integrating all the parameters analyzed in this review.

## 7. Conclusions

In this comprehensive review, we attempt to provide an up-to-date status of the various biomarkers of various origins that may play a role in the evolution of the transplant. As shown in Table 1, the main findings for monitoring serological, cellular and genomic biomarkers in transplantation, computational prediction models and the role of cell-free DNA in transplant outcomes are compiled. 

To identify optimal application scenarios, practitioners’ real-world experiences and advances in the field based on biology and logic, such as early immunosuppression methods, are required. To assist as many patients as possible, evolving standards of care must be used, as well as pushing the boundaries of what is possible, and ddcfDNA is no exception.

ddcfDNA is an exciting new tool in transplantation because of its ability to exploit DNA properties such as specificity and traceability through ancestral lineages, and it is not surprising that we have not figured out how to use it optimally yet. Furthermore, when dealing with unintended consequences for patients, the monetary and other costs of testing must be balanced against the beneficence and nonmaleficence principles. In allogenic transplantation, omics technologies, ddcfDNA analysis and computational model designs are likely to be an integral part of future multifaceted testing strategies such as DSA (both anti-HLA and non-anti-HLA), gene expression (biopsy tissue and peripheral blood), analysis of resolving urinary parameters and biomarkers and other future tests. The main issues may be the absence of these biomarkers in clinical practice, the lack of standardized protocols that allow for intercomparisons and validations and the high cost and time consumption of current detection methods. However, we must incorporate research-based technologies to monitor transplantation’s evolution into our services to provide the best health care to our recipients.

An essential problem in the routine clinical practice of the evolution and results of transplantation, not only in research, is the high economic cost of some of these markers. Some serological biomarkers are non-commercial and economical, depending on having an immunology laboratory with experience in their implementation and standardization. The commercials show affordable prices. Regarding the cost of cellular methods, one must be aware that it will depend on the number of these monoclonal antibody markers (CDs) for flow cytometry. An extensive panel of these markers can make their use expensive in routine clinical practice. These must be truly informative for the evolution of the graft in order to make its implementation viable as a routine.

Regarding genomic and transcriptomic markers, their handicap is their high price, especially gene expression profiles, methylation analyses or miRNA expression. The analysis of circulating DNA is also relatively high-cost for its implementation in routine clinical practice for all patients who want to assess the graft status. In this sense, we believe that basic parameters such as alterations in serological markers that are very easy and relatively cheap to evaluate, either informative cell markers or computational approaches, may precede the use in selected patients of these other much more expensive genomic and transcriptional technologies.

## Figures and Tables

**Table 1:** Compilation of the main findings used for monitoring of serological, cellular and genomic biomarkers in transplantation, computational prediction models and role of cell-free DNA in transplant outcome.

Serological and Urine Biomarkers		Cellular Biomarkers		Genomic and Transcriptomic Biomarkers		Computational Prediction Biomarkers		Cell-Free DNA Biomarker
Classical Markers	New Markers	Classical Markers	New Markers	Classical Markers	New Markers	Classical Markers	New Markers	New Markers
Physical examination	Neutrophil gelatinase-associated lipocalin in urine (uNGAL)	Expression of costimulatory molecules (CD28, CD69, CD95...)	Monitoring of Tregs and Bregs cells post-transplantation	AlloMap and AlloSure assays	AlloMap gene expression profiling test	Rene Dusquesnoy’s HLAmatchmaker tools	Stepwise logistic regression methods	Donor-specific cell-free DNA (ddcfDNA)
Kidney biopsy	Kidney injury molecule-1 (KIM-1)	Production of soluble or intracytoplasmic cytokines	Determination of B cell clusters	Signatera^TM^ and Prospera^TM^ trials	TruGraf^®^ (Transplant Genomics)	Predicted Indirectly Recognizable HLA Epitopes (PIRCHE-II) algorithm	GEO databases, Weighted Gene Coexpression Network Analysis (WGC-NA) algorithm	
Albuminuria	CXCL-10	ImmuKnow assay (Cylex) to assess changes in ATP production by CD4+ T cells	Circulating B cell follicles (cTfh)	Donor plasma mitochondrial DNA (mtDNA)	Viracor TRAC^®^ (Eurofins Viracor)		STRING database	
Proteinuria	Cystatin C (CysC)		B cell differentiation molecules (BR3/BAFF-R, TACI and APRIL)		OmniGrafTM (Transplant Genomics)		DisGeNET	
Determination of serum creatinine level	Osteopontin (OPN)				QSant (Nephrosant) assay		Cytoscape software	
Estimation of glomerular filtration rate (eGFR)	Clusterin (CLU)				miR-142-3p, miR-155, miR-142-5p miR-338-5p miR-150-5p			
HLA antibody profiling	CXCL13							

## Data Availability

All important data is included in the manuscript.

## Abbreviations

AR, acute rejection; AMR, antibody-mediated rejection; cfDNA, cell-free DNA; ddcfDNA, donor-derived cell-free DNA; DSA, donor-specific antibodies; HLA, human leukocyte antigens; HSCT, hematopoietic stem cell transplant; KIR, killer cell immunoglobulin-like receptors; KT, kidney transplant, MM, mismatching; NAR, non-acute rejection; CR, chronic rejection; NCR: non-chronic rejection.

## References

[1] Aldea P.L., Rachisan A.L., Stanciu B.I., Picos A., Picos A.M., Delean D.I., Stroescu R., Starcea M.I., Borzan C.M., Elec F.I. (2022). The Perspectives of Biomarkers in Predicting the Survival of the Renal Graft. Front. Pediatr..

[2] Oellerich M., Budde K., Osmanodja B., Bornemann-Kolatzki K., Beck J., Schütz E., Walson P.D. (2022). Donor-derived cell-free DNA as a diagnostic tool in transplantation. Front. Genet..

[3] Mezzolla V., Pontrelli P., Fiorentino M., Stasi A., Franzin R., Rascio F., Grandaliano G., Stallone G., Infante B., Gesualdo L. (2021). Emerging biomarkers of delayed graft function in kidney transplantation. Transplant. Rev..

[4] DeLuca D.S., Blasczyk R. (2007). HistoCheck. Evaluating structural and functional MHC similarities. Methods Mol. Biol..

[5] Duquesnoy R.J. (2016). Reflections on HLA epitope-based matching for transplantation. Front. Immunol..

[6] Choudhary N.S., Saigal S., Bansal R.K., Saraf N., Gautam D., Soin A.S. (2017). Acute and Chronic Rejection After Liver Transplantation: What A Clinician Needs to Know. J. Clin. Exp. Hepatol..

[7] Pontrelli P., Rascio F., Castellano G., Grandaliano G., Gesualdo L., Stallone G. (2020). The Role of Natural Killer Cells in the Immune Response in Kidney Transplantation. Front. Immunol..

[8] Lemieux W., Fleischer D., Yang A.Y., Niemann M., Oualkacha K., Klement W., Richard L., Polychronakos C., Liwski R., Claas F. (2022). Dissecting the impact of molecular T-cell HLA mismatches in kidney transplant failure: A retrospective cohort study. Front. Immunol..

[9] Boix F., Trujillo C., Muro M. (2018). Cell-Mediated Immunity (CMI) as the Instrument to Assess the Response Against the Allograft: Present and Future. Curr. Protein Pept. Sci..

[10] Chenouard A., Chesneau M., Bui Nguyen L., Le Bot S., Cadoux M., Dugast E., Paul C., Malard-Castagnet S., Ville S., Guérif P. (2017). Renal Operational Tolerance Is Associated with a Defect of Blood Tfh Cells That Exhibit Impaired B Cell Help. Am. J. Transplant..

[11] Cano-Romero F.L., Laguna Goya R., Utrero-Rico A., Gómez-Massa E., Arroyo-Sánchez D., Suárez-Fernández P., Lora D., Andrés A., Castro-Panete M.J., Paz-Artal E. (2019). Longitudinal profile of circulating T follicular helper lymphocytes parallels anti-HLA sensitization in renal transplant recipients. Am. J. Transplant..

[12] Briceño J. (2020). Artificial intelligence and organ transplantation: Challenges and expectations. Curr. Opin. Organ Transplant..

[13] Neupane A.S., Kubes P. (2022). Imaging reveals novel innate immune responses in lung, liver, and beyond. Immunol. Rev..

[14] Demetris A.J., Bellamy C., Hübscher S.G., O’Leary J., Randhawa P.S., Feng S., Neil D., Colvin R.B., McCaughan G., Fung J.J. (2016). 2016 comprehensive update of the Banff working group on liver allograft pathology: Introduction of antibody-mediated rejection. Am. J. Transplant..

[15] Angelico R., Sensi B., Manzia T.M., Tisone G., Grassi G., Signorello A., Milana M., Lenci I., Baiocchi L. (2021). Chronic rejection after liver transplantation: Opening the Pandora’s box. World J. Gastroenterol..

[16] Leibler C., Matignon M., Pilon C., Montespan F., Bigot J., Lang P., Carosella E.D., Cohen J., Rouas-Freiss N., Grimbert P. (2014). Kidney Transplant Recipients Treated with Belatacept Exhibit Increased Naïve and Transitional B Cells. Am. J. Transplant..

[17] Jaikaransingh V., Kadambi P.V. (2021). Donor-derived cell-free DNA (ddcf-DNA) and acute antibody-mediated rejection in kidney transplantation. Medicina.

[18] Zhou Y., Cheng D., Jiang T. (2021). The role of donor-derived cell-free DNA in the detection of renal allograft injury. Nephrol. Ther..

[19] Beck J., Oellerich M., Schulz U., Schauerte V., Reinhard L., Fuchs U., Knabbe C., Zittermann A., Olbricht C., Gummert J.F. (2015). Donor-Derived Cell-Free DNA Is a Novel Universal Biomarker for Allograft Rejection in Solid Organ Transplantation. Transplant. Proc..

[20] Oellerich M., Sherwood K., Keown P., Schütz E., Beck J., Stegbauer J., Rump L.C., Walson P.D. (2021). Liquid biopsies: Donor-derived cell-free DNA for the detection of kidney allograft injury. Nat. Rev. Nephrol..

[21] Crichton E.S., Zeng S., la Muraglia G.M., Badell I.R. (2021). CXCL13 Is an Indicator of Germinal Center Activity and Alloantibody Formation Following Transplantation. Transplant. Direct.

[22] Găman M.A., Cozma M.A., Dobrică E.C., Crețoiu S.M., Găman A.M., Diaconu C.C. (2021). Liquid biopsy and potential liquid biopsy-based biomarkers in philadelphia-negative classical myeloproliferative neoplasms: A systematic review. Life.

[23] Boix F., Millan O., San Segundo D., Mancebo E., Rimola A., Fabrega E., Fortuna V., Mrowiec A., Castro-Panete M.J., de la Peña J. (2016). High expression of CD38, CD69, CD95 and CD154 biomarkers in cultured peripheral T lymphocytes correlates with an increased risk of acute rejection in liver allograft recipients. Immunobiology.

[24] Boix F., Legaz I., Minhas A., Alfaro R., Jiménez–Coll V., Mrowiec A., Martínez–Banaclocha H., Galián J.A., Botella C., Moya–Quiles M.R. (2021). Identification of peripheral CD154+ T cells and HLA-DRB1 as biomarkers of acute cellular rejection in adult liver transplant recipients. Clin. Exp. Immunol..

[25] Boix F., Bolarín J.M., Mrowiec A., Eguía J., Gonzalez-Martinez G., de la Peña J., Galian J.A., Alfaro R., Moya-Quiles M.R., Legaz I. (2017). CD28 biomarker quantification and expression level profiles in CD4+ T-lymphocytes in solid organ transplantation. Transpl. Immunol..

[26] Alfaro R., Legaz I., González-Martínez G., Jimenez-Coll V., Martínez-Banaclocha H., Galián J.A., Botella C., de la Peña-Moral J., Moya-Quiles M.R., Campillo J.A. (2021). Monitoring of b cell in kidney transplantation: Development of a novel clusters analysis and role of transitional b cells in transplant outcome. Diagnostics.

[27] Boix-Giner F., Millan O., San Segundo D., Muñoz-Cacho P., Mancebo E., Llorente S., Rafael-Valdivia L., Rimola A., Fábrega E., Mrowiec A. (2016). High frequency of central memory regulatory T cells allows detection of liver recipients at risk of early acute rejection within the first month after transplantation. Int. Immunol..

[28] Seiler L.K., Phung N.L., Nikolin C., Immenschuh S., Erck C., Kaufeld J., Haller H., Falk C.S., Jonczyk R., Lindner P. (2022). An Antibody-Aptamer-Hybrid Lateral Flow Assay for Detection of CXCL9 in Antibody-Mediated Rejection after Kidney Transplantation. Diagnostics.

[29] López-Álvarez M.R., Moya-Quiles M.R., Minguela A., Gil J., Miras M., Campillo J.A., Díaz-Alderete M.A., García-Alonso A.M., Sánchez-Bueno F., Vicario J.L. (2009). HLA-C matching and liver transplants: Donor-recipient genotypes influence early outcome and CD8+KIR2D+ T-cells recuperation. Transplantation.

[30] Heyne N., Kemmner S., Schneider C., Nadalin S., Königsrainer A., Häring H.U. (2012). Urinary neutrophil gelatinase-associated lipocalin accurately detects acute allograft rejection among other causes of acute kidney injury in renal allograft recipients. Transplantation.

[31] He J., Tsai L.M., Leong Y.A., Hu X., Ma C.S., Chevalier N., Sun X., Vandenberg K., Rockman S., Ding Y. (2013). Circulating Precursor CCR7loPD-1hi CXCR5+ CD4+ T Cells Indicate Tfh Cell Activity and Promote Antibody Responses upon Antigen Reexposure. Immunity.

[32] El band J.E.K., Llorente S., Martinez-Garcia P., Alfaro R., Jimenez-Coll V., Boix F., Galián J.A., Martinez-Banaclocha H., Botella C., Moya-Quiles M.R. (2021). Evaluation of Antibodies Directed Against Two GPCRs, Anti-AT1R and Anti-ETAR, on Kidney Transplant Outcome. Curr. Protein Pept. Sci..

[33] Alfaro R., Jaouad E.K.E.B., Llorente S., Jimenez-Coll V., Martínez-Banaclocha H., Galián J.A., Botella C., Moya-Quiles M.R., Peña-Moral J.D.L., Minguela A. (2021). Personalized Medicine for Kidney Transplantation: Association of Graft Survival and Acute Transplant Rejection with Genetic Variation in B Cell Activating Factor System Signaling. OMICS A J. Integr. Biol..

[34] Alfaro R., Legaz I., Jimenez-Coll V., El Kaaoui El Band J., Martínez-Banaclocha H., Galián J.A., Parrado A., Mrowiec A., Botella C., Moya-Quiles M.R. (2021). MicroRNA Expression Changes in Kidney Transplant: Diagnostic Efficacy of miR-150-5p as Potential Rejection Biomarker, Pilot Study. J. Clin. Med..

[35] De Vlaminck I., Martin L., Kertesz M., Patel K., Kowarsky M., Strehl C., Cohen G., Luikart H., Neff N.F., Okamoto J. (2015). Noninvasive monitoring of infection and rejection after lung transplantation. Proc. Natl. Acad. Sci. USA.

[36] Pattar S., Aleinati M., Iqbal F., Madhu A., Blais S., Wang X., Dallaire F., Wang Y., Isaac D., Fine N. (2021). Identification of cell-free DNA methylation patterns unique to the human left ventricle as a potential indicator of acute cellular rejection. Clin. Transplant..

[37] Cristoferi I., Giacon T.A., Boer K., van Baardwijk M., Neri F., Campisi M., Kimenai H.J.A.N., Clahsen - van Groningen M.C., Pavanello S., Furian L. (2022). The applications of DNA methylation as a biomarker in kidney transplantation: A systematic review. Clin. Epigenetics.

[38] Silverman E.K., Schmidt H.H.H.W., Anastasiadou E., Altucci L., Angelini M., Badimon L., Balligand J.L., Benincasa G., Capasso G., Conte F. (2020). Molecular networks in Network Medicine: Development and applications. Wiley Interdiscip. Rev. Syst. Biol. Med..

[39] Rabant M., Amrouche L., Lebreton X., Aulagnon F., Benon A., Sauvaget V., Bonifay R., Morin L., Scemla A., Delville M. (2015). Urinary C-X-C Motif Chemokine 10 Independently Improves the Noninvasive Diagnosis of Antibody–Mediated Kidney Allograft Rejection. J. Am. Soc. Nephrol..

[40] Weseslindtner L., Hedman L., Wang Y., Strassl R., Helanterä I., Aberle S.W., Bond G., Hedman K. (2020). Longitudinal assessment of the CXCL10 blood and urine concentration in kidney transplant recipients with BK polyomavirus replication—a retrospective study. Transpl. Int..

[41] Tatapudi R.R., Muthukumar T., Dadhania D., Ding R., Li B., Sharma V.K., Lozada-Pastorio E., Seetharamu N., Hartono C., Serur D. (2004). Noninvasive detection of renal allograft inflammation by measurements of mRNA for IP-10 and CXCR3 in urine. Kidney Int..

[42] Lazzeri E., Rotondi M., Mazzinghi B., Lasagni L., Buonamano A., Rosati A., Pradella F., Fossombroni V., La Villa G., Gacci M. (2005). High CXCL10 expression in rejected kidneys and predictive role of pretransplant serum CXCL10 for acute rejection and chronic allograft nephropathy. Transplantation.

[43] Jackson J.A., Kim E.J., Begley B., Cheeseman J., Harden T., Perez S.D., Thomas S., Warshaw B., Kirk A.D. (2011). Urinary Chemokines CXCL9 and CXCL10 Are Noninvasive Markers of Renal Allograft Rejection and BK Viral Infection. Am. J. Transplant..

[44] Merhi B., Bayliss G., Gohh R.Y. (2015). Role for urinary biomarkers in diagnosis of acute rejection in the transplanted kidney. World J. Transplant..

[45] Mussap M., Plebani M. (2008). Biochemistry and Clinical Role of Human Cystatin C. Crit. Rev. Clin. Lab. Sci..

[46] Jensen D., Kierulf-Lassen C., Kristensen M.L.V., Nørregaard R., Weyer K., Nielsen R., Christensen E.I., Birn H. (2017). Megalin dependent urinary cystatin C excretion in ischemic kidney injury in rats. PLoS ONE.

[47] Szirmay B., Kustán P., Horváth-Szalai Z., Ludány A., Lakatos Á., Mühl D., Wittmann I., Miseta A., Kovács G.L., Koszegi T. (2018). Novel automated immune turbidimetric assay for routine urinary cystatin-C determinations. Bioanalysis.

[48] Spahillari A., Parikh C.R., Sint K., Koyner J.L., Patel U.D., Edelstein C.L., Passik C.S., Thiessen-Philbrook H., Swaminathan M., Shlipak M.G. (2012). Serum Cystatin C– Versus Creatinine-Based Definitions of Acute Kidney Injury Following Cardiac Surgery: A Prospective Cohort Study. Am. J. Kidney Dis..

[49] Taghizadeh-Afshari A., Mohammadi-Fallah M., Alizadeh M., Abkhiz S., Valizadeh R., Khadem-Ansari M.H., Sayyadi H., Kashani S.A., Rahimi M.M. (2017). Serum cystatin C versus creatinine in the assessment of allograft function in early periods of kidney transplantation. J. Ren. Inj. Prev..

[50] Higashi A., Dohi Y., Uraoka N., Sentani K., Uga S., Kinoshita H., Sada Y., Kitagawa T., Hidaka T., Kurisu S. (2015). The Potential Role of Inflammation Associated with Interaction between Osteopontin and CD44 in a Case of Pulmonary Tumor Thrombotic Microangiopathy Caused by Breast Cancer. Intern. Med..

[51] Millán O., Rafael-Valdivia L., San Segundo D., Boix F., Castro-Panete M.J., López-Hoyos M., Muro M., Valero-Hervás D., Rimola A., Navasa M. (2014). Should IFN-γ, IL-17 and IL-2 be considered predictive biomarkers of acute rejection in liver and kidney transplant? Results of a multicentric study. Clin. Immunol..

[52] Askenazi D.J., Koralkar R., Hundley H.E., Montesanti A., Parwar P., Sonjara S., Ambalavanan N. (2012). Urine Biomarkers Predict Acute Kidney Injury in Newborns. J. Pediatr..

[53] Castello L.M., Raineri D., Salmi L., Clemente N., Vaschetto R., Quaglia M., Garzaro M., Gentilli S., Navalesi P., Cantaluppi V. (2017). Osteopontin at the Crossroads of Inflammation and Tumor Progression. Mediators Inflamm..

[54] Alchi B., Nishi S., Kondo D., Kaneko Y., Matsuki A., Imai N., Ueno M., Iguchi S., Sakatsume M., Narita I. (2005). Osteopontin expression in acute renal allograft rejection. Kidney Int..

[55] Wang J., Tang Q., Qiu Y., Xu M., Rong R., Zhu T. (2013). Osteopontin level correlates with acute cellular renal allograft rejection. J. Surg. Res..

[56] Zhou W., Guan Q., Kwan C.C.H., Chen H., Gleave M.E., Nguan C.Y.C., Du C. (2010). Loss of clusterin expression worsens renal ischemia-reperfusion injury. Am. J. Physiol.-Ren. Physiol..

[57] Pezeshgi A., Azar S.A., Ghasemi H., Kamali K., Esmaeilzadeh A., Hajsalimi B., Pour-Asghar S., Behmanesh M.R., Kiafar M. (2016). Role of plasma neutrophil gelatinase-associated lipocalin as an emerging biomarker of acute renal failure following kidney transplantation and its correlation with plasma creatinine. J. Ren. Inj. Prev..

[58] Wu C.Y., Yang H.Y., Chien H.P., Tseng M.H., Huang J.L. (2018). Urinary clusterin—A novel urinary biomarker associated with pediatric lupus renal histopathologic features and renal survival. Pediatr. Nephrol..

[59] Musiał K., Augustynowicz M., Miśkiewicz-Migoń I., Kałwak K., Ussowicz M., Zwolińska D. (2020). Clusterin as a New Marker of Kidney Injury in Children Undergoing Allogeneic Hematopoietic Stem Cell Transplantation—A Pilot Study. J. Clin. Med..

[60] Pianta T.J., Peake P.W., Pickering J.W., Kelleher M., Buckley N.A., Endre Z.H. (2015). Clusterin in kidney transplantation: Novel biomarkers versus serum creatinine for early prediction of delayed graft function. Transplantation.

[61] San Segundo D., Millán O., Munoz-Cacho P., Boix F., Paz-Artal E., Talayero P., Morales J.M., Muro M., De Cos M.Á., Guirado L. (2014). High proportion of pretransplantation activated regulatory T cells (CD4+ CD25highCD62L+ CD45RO+) predicts acute rejection in kidney transplantation: Results of a multicenter study. Transplantation.

[62] Blanco-García R.M., López-Álvarez M.R., Garrido I.P., Salgado-Cecilia G., Campillo J.A., Bolarín J.M., Legaz I., Muro M., García-Alonso A.M., Martínez-Sánchez M.V. (2011). CD28 and KIR2D receptors as sensors of the immune status in heart and liver transplantation. Hum. Immunol..

[63] Laguna-Goya R., Utrero-Rico A., Cano-Romero F.L., Gómez-Massa E., González E., Andrés A., Mancebo-Sierra E., Paz-Artal E. (2020). Imbalance favoring follicular helper T cells over IL10+ regulatory B cells is detrimental for the kidney allograft. Kidney Int..

[64] Achour A., Simon Q., Mohr A., Séité J.F., Youinou P., Bendaoud B., Ghedira I., Pers J.O., Jamin C. (2017). Human regulatory B cells control the TFH cell response. J. Allergy Clin. Immunol..

[65] O’Halloran C., Cullen K., Njoroge J., Jessop L., Smith J., Hope V., Ncube F. (2017). The extent of and factors associated with self-reported overdose and self-reported receipt of naloxone among people who inject drugs (PWID) in England, Wales and Northern Ireland. Int. Jounal Drug Policy.

[66] Xu Y., Zhang H., Zhang D., Wang Y., Wang Y., Wang W., Hu X. (2022). Identification of a novel peripheral blood signature diagnosing subclinical acute rejection after renal transplantation. Transl. Androl. Urol..

[67] Halloran P.F., Reeve J., Madill-Thomsen K.S., Kaur N., Ahmed E., Cantos C., Al Haj Baddar N., Demko Z., Liang N., Swenerton R.K. (2022). Combining Donor-derived Cell-free DNA Fraction and Quantity to Detect Kidney Transplant Rejection Using Molecular Diagnoses and Histology as Confirmation. Transplantation.

[68] Benincasa G., Viglietti M., Coscioni E., Napoli C. (2022). “Transplantomics” for predicting allograft rejection: Real-life applications and new strategies from Network Medicine. Hum. Immunol..

[69] Seeto R.K., Fleming J.N., Dholakia S., Dale B.L. (2020). Understanding and using AlloSure donor derived cell-free DNA. Biophys. Rev..

[70] Han F., Sun Q., Huang Z., Li H., Ma M., Liao T., Luo Z., Zheng L., Zhang N., Chen N. (2021). Donor plasma mitochondrial DNA is associated with antibody-mediated rejection in renal allograft recipients. Aging (Albany NY).

[71] Loupy A., Duong Van Huyen J.P., Hidalgo L., Reeve J., Racapé M., Aubert O., Venner J.M., Falmuski K., Cécile Bories M., Beuscart T. (2017). Gene expression profiling for the identification and classification of antibody-mediated heart rejection. Circulation.

[72] Warmuzińska N., Łuczykowski K., Bojko B. (2022). A Review of Current and Emerging Trends in Donor Graft-Quality Assessment Techniques. J. Clin. Med..

[73] Halloran P.F., Reeve J., Akalin E., Aubert O., Bohmig G.A., Brennan D., Bromberg J., Einecke G., Eskandary F., Gosset C. (2017). Real Time Central Assessment of Kidney Transplant Indication Biopsies by Microarrays: The INTERCOMEX Study. Am. J. Transplant..

[74] Eller K., Böhmig G.A., Banas M.C., Viklicky O. (2022). Editorial: Advances in the diagnosis and treatment in kidney transplantation. Front. Med..

[75] Halloran P.F., Pereira A.B., Chang J., Matas A., Picton M., De Freitas D., Bromberg J., Serõn D., Sellarés J., Einecke G. (2013). Microarray Diagnosis of Antibody-Mediated Rejection in Kidney Transplant Biopsies: An International Prospective Study (INTERCOM). Am. J. Transplant..

[76] Anglicheau D., Sharma V.K., Ding R., Hummel A., Snopkowski C., Dadhania D., Seshan S.V., Suthanthiran M. (2009). MicroRNA expression profiles predictive of human renal allograft status. Proc. Natl. Acad. Sci. USA.

[77] Ben-Dov I.Z., Muthukumar T., Morozov P., Mueller F.B., Tuschl T., Suthanthiran M. (2012). MicroRNA Sequence Profiles of Human Kidney Allografts With or Without Tubulointerstitial Fibrosis. Transplantation.

[78] Flower D.R. (2007). Immunoinformatics and the in silico prediction of immunogenicity. An introduction. Methods Mol. Biol..

[79] Burlacu A., Iftene A., Jugrin D., Popa I.V., Lupu P.M., Vlad C., Covic A. (2020). Using Artificial Intelligence Resources in Dialysis and Kidney Transplant Patients: A Literature Review. Biomed Res. Int..

[80] Topuz K., Zengul F.D., Dag A., Almehmi A., Yildirim M.B. (2018). Predicting graft survival among kidney transplant recipients: A Bayesian decision support model. Decis. Support Syst..

[81] Shaikhina T., Lowe D., Daga S., Briggs D., Higgins R., Khovanova N. (2019). Decision tree and random forest models for outcome prediction in antibody incompatible kidney transplantation. Biomed. Signal Process. Control.

[82] Segev D.L., Gentry S.E., Warren D.S., Reeb B., Montgomery R.A. (2005). Kidney Paired Donation and Optimizing the Use of Live Donor Organs. JAMA.

[83] Patterson E.A., Whelan M.P. (2017). A framework to establish credibility of computational models in biology. Prog. Biophys. Mol. Biol..

[84] Narayan A., Liu Z., Bergquist J.A., Charlebois C., Rampersad S., Rupp L., Brooks D., White D., Tate J., MacLeod R.S. (2023). UncertainSCI: Uncertainty quantification for computational models in biomedicine and bioengineering. Comput. Biol. Med..

[85] Alfaro R., Martínez-Banaclocha H., Llorente S., Jimenez-Coll V., Galián J.A., Botella C., Moya-Quiles M.R., Parrado A., Muro-Perez M., Minguela A. (2021). Computational Prediction of Biomarkers, Pathways, and New Target Drugs in the Pathogenesis of Immune-Based Diseases Regarding Kidney Transplantation Rejection. Front. Immunol..

[86] Agapito G., Cannataro M. A parallel software pipeline to select relevant genes for pathway enrichment. Proceedings of the 2022 30th Euromicro International Conference on Parallel, Distributed and Network-based Processing (PDP).

[87] Maron B.A., Wang R.S., Shevtsov S., Drakos S.G., Arons E., Wever-Pinzon O., Huggins G.S., Samokhin A.O., Oldham W.M., Aguib Y. (2021). Individualized interactomes for network-based precision medicine in hypertrophic cardiomyopathy with implications for other clinical pathophenotypes. Nat. Commun..

[88] Hartzell S., Bin S., Cantarelli C., Haverly M., Manrique J., Angeletti A., Manna G.L., Murphy B., Zhang W., Levitsky J. (2020). Kidney Failure Associates with T Cell Exhaustion and Imbalanced Follicular Helper T Cells. Front. Immunol..

[89] Wang L.J., Ma X.B., Xia H.Y., Sun X., Yu L., Yang Q., Hu Z.Q., Zhao Y.H., Hu W., Ran J.H. (2021). Identification of Biomarkers for Predicting Allograft Rejection following Kidney Transplantation Based on the Weighted Gene Coexpression Network Analysis. Biomed Res. Int..

[90] Teng L., Shen L., Zhao W., Wang C., Feng S., Wang Y., Bi Y., Rong S., Shushakova N., Haller H. (2022). SLAMF8 Participates in Acute Renal Transplant Rejection *via* TLR4 Pathway on Pro-Inflammatory Macrophages. Front. Immunol..

[91] Benincasa G., Maron B.A., Affinito O., D’Alto M., Franzese M., Argiento P., Schiano C., Romeo E., Bontempo P., Golino P. (2022). Association Between Circulating CD4+ T Cell Methylation Signatures of Network-Oriented SOCS3 Gene and Hemodynamics in Patients Suffering Pulmonary Arterial Hypertension. J. Cardiovasc. Transl. Res..

[92] Legaz I., Bernardo M.V., Alfaro R., Martínez-Banaclocha H., Galián J.A., Jimenez-Coll V., Boix F., Mrowiec A., Salmeron D., Botella C. (2021). PCR Array Technology in Biopsy Samples Identifies Up-Regulated mTOR Pathway Genes as Potential Rejection Biomarkers After Kidney Transplantation. Front. Med..

[93] Snyder T.M., Khush K.K., Valantine H.A., Quake S.R. (2011). Universal noninvasive detection of solid organ transplant rejection. Proc. Natl. Acad. Sci. USA.

[94] STRING: Functional Protein Association Networks. https://string-db.org/.

[95] Starzl T.E., Murase N., Ildstad S., Ricordi C., Demetris A.J., Trucco M. (1992). Cell migration, chimerism, and graft acceptance. Lancet.

[96] Lo Y.M.D., Tein M.S.C., Pang C.C.P., Yeung C.K., Tong K.L., Magnus Hjelm N. (1998). Presence of donor-specific DNA in plasma of kidney and liver-transplant recipients. Lancet.

[97] Liu Z., Zhao J., Wang W., Zhu H., Qian J., Wang S., Que S., Zhang F., Yin S., Zhou L. (2021). Integrative Network Analysis Revealed Genetic Impact of Pyruvate Kinase L/R on Hepatocyte Proliferation and Graft Survival after Liver Transplantation. Oxid. Med. Cell. Longev..

[98] Gadi V.K., Nelson J.L., Boespflug N.D., Guthrie K.A., Kuhr C.S. (2006). Soluble Donor DNA Concentrations in Recipient Serum Correlate with Pancreas-Kidney Rejection. Clin. Chem..

[99] Goussous N., Xie W., Dawany N., Scalea J.R., Bartosic A., Haririan A., Kalil R., Drachenberg C., Costa N., Weir M.R. (2020). Donor-derived Cell-free DNA in Infections in Kidney Transplant Recipients: Case Series. Transplant. Direct.

[100] Kant S., Bromberg J., Haas M., Brennan D. (2020). Donor-derived Cell-free DNA and the Prediction of BK Virus-associated Nephropathy. Transplant. Direct.

[101] Sigdel T.K., Archila F.A., Constantin T., Prins S.A., Liberto J., Damm I., Towfighi P., Navarro S., Kirkizlar E., Demko Z.P. (2018). Optimizing Detection of Kidney Transplant Injury by Assessment of Donor-Derived Cell-Free DNA via Massively Multiplex PCR. J. Clin. Med..

[102] Jordan S.C., Bunnapradist S., Bromberg J.S., Langone A.J., Hiller D., Yee J.P., Sninsky J.J., Woodward R.N., Matas A.J. (2018). Donor-derived Cell-free DNA Identifies Antibody-mediated Rejection in Donor Specific Antibody Positive Kidney Transplant Recipients. Transplant. Direct.

[103] Huang E., Sethi S., Peng A., Najjar R., Mirocha J., Haas M., Vo A., Jordan S.C. (2019). Early clinical experience using donor-derived cell-free DNA to detect rejection in kidney transplant recipients. Am. J. Transplant..

